# Retracted: Decursin Isolated from* Angelica gigas* Nakai Rescues PC12 Cells from Amyloid *β*-Protein-Induced Neurotoxicity through Nrf2-Mediated Upregulation of Heme Oxygenase-1: Potential Roles of MAPK

**DOI:** 10.1155/2019/2986594

**Published:** 2019-01-02

**Authors:** Evidence-Based Complementary and Alternative Medicine

At the request of the authors, the article titled “Decursin Isolated from Angelica gigas Nakai Rescues PC12 Cells from Amyloid *β*-Protein-Induced Neurotoxicity through Nrf2-Mediated Upregulation of Heme Oxygenase-1: Potential Roles of MAPK” [1] has been retracted. The article was found to have serious concerns in the figures. An erratum [2] was previously published to correct Figures 2(a) and 5(b), but an additional figure duplication issue was raised on PubPeer in which the GADPH bands in Figure 6(a) are the same as those in Figure 6(c) but rotated 180 degrees.

We asked the authors to provide us with the raw blots for every figure in the original article and the erratum, but they did not have the original data because the experiments were done ten years ago. The authors said it would take a long time to repeat the results and although they do not believe this affects the conclusions, the authors asked to retract the article to avoid misleading readers. They sincerely apologize for the mistake and inconvenience.
